# 4-Methyl-*N*-[2-(pyridin-2-yl)ethyl­carbamo­thio­yl]benzamide

**DOI:** 10.1107/S1600536814016377

**Published:** 2014-07-31

**Authors:** Farook Adam, Nadiah Ameram, Naser Eltaher Eltayeb

**Affiliations:** a School of Chemical Sciences, 11800, USM Pulau Pinang, Malaysia; b College of Sciences and Arts, Rabigh, King Abdulaziz University, Saudi Arabia

**Keywords:** crystal structure, hydrogen bonding, thio­urea compounds, thio­carbonyl groups, benzamide

## Abstract

In the title compound, C_16_H_17_N_3_OS, the dihedral angle between the planes of the benzene and pyridine rings is 71.33 (15)°. An intra­molecular N—H⋯O hydrogen bond is present. In the crystal, weak aromatic C—H⋯O hydrogen bonds link the mol­ecules into chains extending along *a*.

## Related literature   

For related structures, see: Saeed & Flörke (2007 ▶); Yusof *et al.* (2008 ▶, 2011 ▶); Shoukat *et al.* (2007 ▶); Hassan *et al.* (2008*a* ▶,*b* ▶,*c* ▶). For standard bond lengths, see: Allen *et al.* (1987 ▶). For graph-set analysis, see Bernstein *et al.* (1995 ▶).
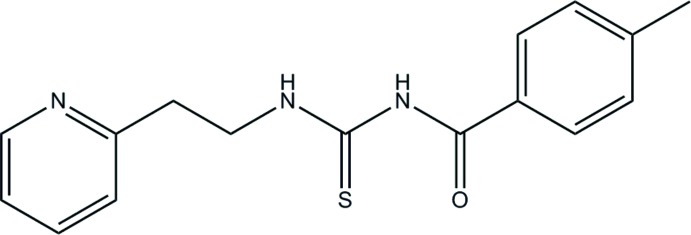


## Experimental   

### 

#### Crystal data  

C_16_H_17_N_3_OS
*M* *_r_* = 299.39
Monoclinic, 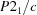

*a* = 16.0467 (12) Å
*b* = 4.8824 (4) Å
*c* = 23.0403 (18) Å
β = 124.997 (5)°
*V* = 1478.7 (2) Å^3^
*Z* = 4
Mo *K*α radiation
μ = 0.22 mm^−1^
*T* = 100 K
0.47 × 0.20 × 0.14 mm

#### Data collection  

Bruker APEXII CCD diffractometer
Absorption correction: multi-scan (*SADABS*; Bruker, 2005 ▶) *T*_min_ = 0.903, *T*_max_ = 0.980
12777 measured reflections
3409 independent reflections
2221 reflections with *I* > 2σ(*I*)
*R*_int_ = 0.082

#### Refinement  

*R*[*F* ^2^ > 2σ(*F* ^2^)] = 0.068
*wR*(*F* ^2^) = 0.182
*S* = 1.04
3409 reflections
199 parameters
H atoms treated by a mixture of independent and constrained refinement
Δρ_max_ = 0.66 e Å^−3^
Δρ_min_ = −0.44 e Å^−3^

### 

Data collection: *APEX2* (Bruker, 2005 ▶); cell refinement: *SAINT* (Bruker, 2005 ▶); data reduction: *SAINT*; program(s) used to solve structure: *SHELXTL* (Sheldrick, 2008 ▶); program(s) used to refine structure: *SHELXTL*; molecular graphics: *SHELXTL*; software used to prepare material for publication: *SHELXTL* and *PLATON* (Spek, 2009 ▶).

## Supplementary Material

Crystal structure: contains datablock(s) I. DOI: 10.1107/S1600536814016377/zs2306sup1.cif

Structure factors: contains datablock(s) I. DOI: 10.1107/S1600536814016377/zs2306Isup2.hkl

Supporting information file. DOI: 10.1107/S1600536814016377/zs2306Isup3.cml

CCDC reference: 1014035

Additional supporting information:  crystallographic information; 3D view; checkCIF report

## Figures and Tables

**Table 1:** Hydrogen-bond geometry (Å, °)

*D*—H⋯*A*	*D*—H	H⋯*A*	*D*⋯*A*	*D*—H⋯*A*
N2—H1N2⋯O2	0.87 (4)	1.90 (3)	2.645 (3)	143 (3)
C14—H14*A*⋯O2^i^	0.95	2.51	3.421 (4)	161

Symmetry code: (i) 
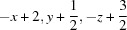
.

**Fig. 1.**
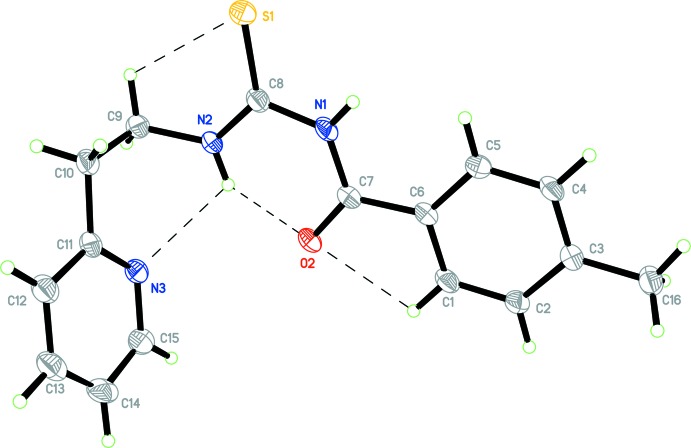
The molecular structure of the title compound, showing 50% probability displacement ellipsoids and the atom-numbering scheme. Intramolecular interactions are shown as dashed lines.

**Fig. 2.**
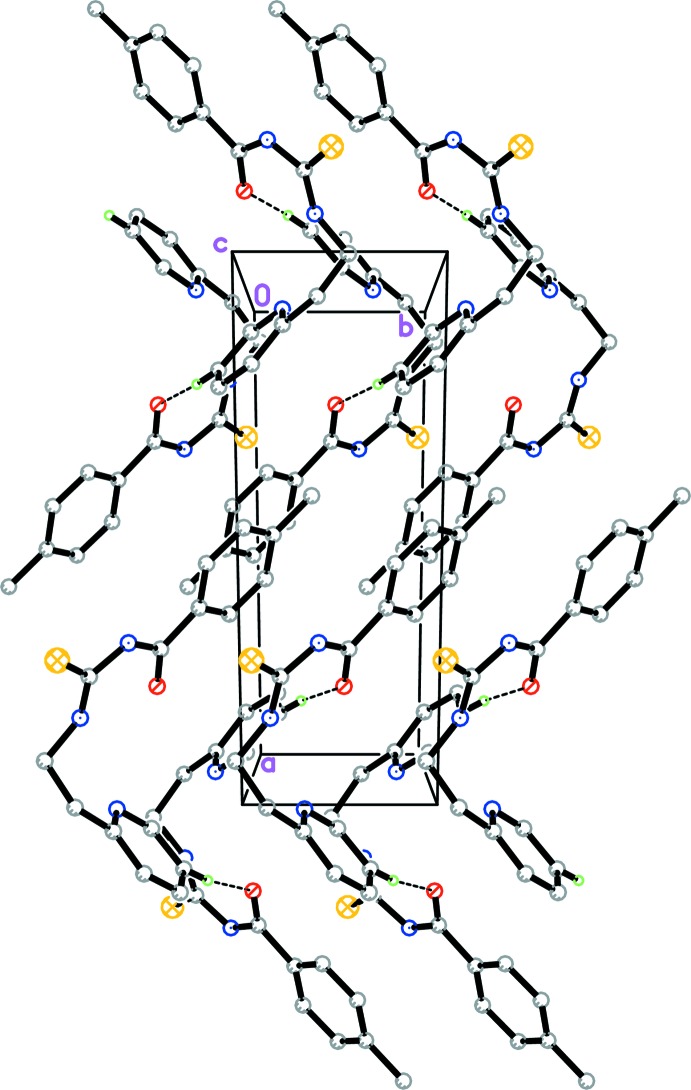
The crystal packing of the title compound viewed down the c axis. Hydrogen bonds are shown as dashed lines.

## supplementary crystallographic information

### S1. Comment

In the title compound, C_16_H_17_N_3_SO (Fig. 1), the bond lengths and angles are generally normal compared to those in *N*-alkyl-*N*-benzoyl­thio­urea compounds (Allen *et al.*, 1987). The bond lengths of the carbonyl and thio­carbonyl groups [C7—O2 = 1.229 (5) Å and C8—S1 = 1.677 (4) Å, respectively] have typical C═O and C═S double-bond character (Yusof *et al.* 2011). However, the thio­carbonyl is longer compared to the typical C═S bond which is 1.660 (2) Å. The C—N bond lengths for the title compound [C7—N1 = 1.375 (4) Å, C8—N1 = 1.397 (4) Å, C9—N2 = 1.460 (4) Å, C11—N3 = 1.335 (4), C15—N3 = 1.351 (4) Å] are all shorter than the average C—N single bond length [1.472 (5) Å], thus showing varying degrees of single bond character (Yusof *et al.* 2008). These bond features in the structure are presumed as a result of the intra­molecular H-bonding inter­actions "locking" the molecule into a planar six-membered ring structure and are consistent with the expected delocalization in the title compound, confimed by the C9—N2—C8 and C8—N1—C7 bond angles [125.0 (3) and 128.8 (3)°, respectively], showing sp^2^ hybridization on the N2 and N1 atoms. The molecule maintains its *cis–trans* configuration with respect to the position of the methyl benzene and ethyl pyridine groups relative to the thio­carbonyl sulfur atom across the N1—C7 and N2—C8 bonds, respectively (Hassan *et al.* (2008*b*,2008*c*)). The conformation of the molecule with respect to the thio­carbonyl and carbonyl moieties is twisted, as refleced by the torsion angles [C8–N1–C7–O2, C7–N1–C8–N2 and C7—N1—C8—S1: 2.1 (5), -4.4 (4) and 175.9 (2)°, respectively. The angle between the benzene and pyridine rings is 71.33 (15)°. The N2 H-atom forms bifurcated intra­molecular inter­actions with both a carbonyl O-atom and the pyridine N-atom (Table 1): a hydrogen bond with O2 (N2—H···O2) and an inter­action with N3 (N2—H···N3), giving cyclic motifs [graph sets *S*6 (Bernstein *et al.*, 1995)]. Present also are weak intra­molecular C1—H···O2 and C9—H···S1 inter­actions [graph set *S*(5)]. In the crystal, molecules are connected through weak inter­molecular C14—H···O2 hydrogen-bonding inter­actions, giving one-dimensional chain structures extending along *x* (Fig. 2). The N1—H1N1 group has no acceptor in the crystal.

### S2.1. Synthesis and crystallization

Freshly prepared substituted *p*-benzoyl chloride (13 mmol) was added dropwise to a stirred acetone solution (30 ml) of ammonium thio­cyanate (13 mmol). The mixture was stirred for 10 min. A solution of 2-(2-amine­thyl­pyridine) in acetone was added and the reaction mixture was refluxed for 3 h., after which the solution was poured into a beaker containing some ice cubes. The resulting precipitate was collected by filtration, washed several times with a cold ethanol/water mixture and purified by recrystallization from ethanol (Hassan *et al.*, 2008*a*). Yield 65%; white transparent crystals, m.p. 126.3 °C. Anal Calc. for C16 H17 N3 O S: C, 64.9; H, 5.6; N, 15.9; S, 8.2%. Found: C, 64.8; H, 5.7; N, 14.8; S, 8.7%.

### S2.2. Refinement

The H-atoms on the N atoms were located in a difference-Fourier and were fully refined. All other H-atoms were positioned geometrically and refined using a riding model with C—H = 0.95–0.99 Å and *U*_iso_(H) = 1.2*U*_eq_(aromatic C), 1.5*U*_eq_(methyl C) and 1.2*U*_eq_(methyl­ene C).

### Crystal data

**Table:**

C_16_H_17_N_3_OS	*F*(000) = 632
*M**_r_* = 299.39	*D*_x_ = 1.345 Mg m^−^^3^
Monoclinic, *P*2_1_/*c*	Melting point: 399.3 K
Hall symbol: -P 2ybc	Mo *K*α radiation, λ = 0.71073 Å
*a* = 16.0467 (12) Å	θ = 2.6–25.5°
*b* = 4.8824 (4) Å	µ = 0.22 mm^−^^1^
*c* = 23.0403 (18) Å	*T* = 100 K
β = 124.997 (5)°	Block, colourless
*V* = 1478.7 (2) Å^3^	0.47 × 0.20 × 0.14 mm
*Z* = 4	

### Data collection

**Table:**

Bruker APEXII CCD diffractometer	3409 independent reflections
Radiation source: fine-focus sealed tube	2221 reflections with *I* > 2σ(*I*)
Graphite monochromator	*R*_int_ = 0.082
φ and ω scans	θ_max_ = 27.6°, θ_min_ = 2.2°
Absorption correction: multi-scan (*SADABS*; Bruker, 2005)	*h* = −17→20
*T*_min_ = 0.903, *T*_max_ = 0.980	*k* = −6→6
12777 measured reflections	*l* = −30→24

### Refinement

**Table:**

Refinement on *F*^2^	Primary atom site location: structure-invariant direct methods
Least-squares matrix: full	Secondary atom site location: difference Fourier map
*R*[*F*^2^ > 2σ(*F*^2^)] = 0.068	Hydrogen site location: inferred from neighbouring sites
*wR*(*F*^2^) = 0.182	H atoms treated by a mixture of independent and constrained refinement
*S* = 1.04	*w* = 1/[σ^2^(*F*_o_^2^) + (0.0899*P*)^2^] where *P* = (*F*_o_^2^ + 2*F*_c_^2^)/3
3409 reflections	(Δ/σ)_max_ < 0.001
199 parameters	Δρ_max_ = 0.66 e Å^−^^3^
0 restraints	Δρ_min_ = −0.44 e Å^−^^3^

### Special details

**Table:**

Geometry. All e.s.d.'s (except the e.s.d. in the dihedral angle between two l.s. planes) are estimated using the full covariance matrix. The cell e.s.d.'s are taken into account individually in the estimation of e.s.d.'s in distances, angles and torsion angles; correlations between e.s.d.'s in cell parameters are only used when they are defined by crystal symmetry. An approximate (isotropic) treatment of cell e.s.d.'s is used for estimating e.s.d.'s involving l.s. planes.
Refinement. Refinement of *F*^2^ against ALL reflections. The weighted *R*-factor *wR* and goodness of fit *S* are based on *F*^2^, conventional *R*-factors *R* are based on *F*, with *F* set to zero for negative *F*^2^. The threshold expression of *F*^2^ > σ(*F*^2^) is used only for calculating *R*-factors(gt) *etc*. and is not relevant to the choice of reflections for refinement. *R*-factors based on *F*^2^ are statistically about twice as large as those based on *F*, and *R*- factors based on ALL data will be even larger.

### Fractional atomic coordinates and isotropic or equivalent isotropic displacement parameters (Å^2^)

**Table:**

	*x*	*y*	*z*	*U*_iso_*/*U*_eq_	
S1	0.72652 (6)	0.04528 (17)	0.87044 (4)	0.0286 (3)	
O2	0.78657 (14)	0.5226 (4)	0.72802 (11)	0.0272 (5)	
N1	0.69712 (19)	0.3898 (5)	0.77218 (13)	0.0227 (6)	
N2	0.84392 (17)	0.1337 (5)	0.82384 (13)	0.0211 (5)	
N3	1.02858 (17)	0.3126 (5)	0.84401 (12)	0.0229 (6)	
C1	0.6442 (2)	0.9043 (6)	0.63777 (15)	0.0236 (7)	
H1A	0.7034	0.8759	0.6386	0.028*	
C2	0.5737 (2)	1.0965 (6)	0.59246 (15)	0.0250 (7)	
H2A	0.5853	1.1996	0.5627	0.030*	
C3	0.4852 (2)	1.1422 (6)	0.58956 (15)	0.0227 (6)	
C4	0.4718 (2)	0.9901 (6)	0.63468 (16)	0.0246 (7)	
H4A	0.4128	1.0199	0.6341	0.030*	
C5	0.5426 (2)	0.7954 (6)	0.68073 (15)	0.0245 (7)	
H5A	0.5315	0.6933	0.7108	0.029*	
C6	0.6301 (2)	0.7508 (6)	0.68249 (14)	0.0200 (6)	
C7	0.7113 (2)	0.5479 (6)	0.72911 (14)	0.0203 (6)	
C8	0.7611 (2)	0.1903 (6)	0.82127 (15)	0.0218 (6)	
C9	0.9261 (2)	−0.0462 (6)	0.87572 (15)	0.0240 (7)	
H9A	0.9508	−0.1556	0.8523	0.029*	
H9B	0.9001	−0.1743	0.8952	0.029*	
C10	1.0135 (2)	0.1207 (6)	0.93572 (15)	0.0242 (7)	
H10A	0.9874	0.2330	0.9578	0.029*	
H10B	1.0650	−0.0066	0.9723	0.029*	
C11	1.0649 (2)	0.3085 (6)	0.91261 (15)	0.0224 (6)	
C12	1.1454 (2)	0.4739 (6)	0.96253 (16)	0.0282 (7)	
H12A	1.1707	0.4633	1.0112	0.034*	
C13	1.1880 (2)	0.6550 (7)	0.93981 (18)	0.0315 (8)	
H13A	1.2426	0.7710	0.9728	0.038*	
C14	1.1503 (2)	0.6643 (6)	0.86918 (18)	0.0323 (8)	
H14A	1.1775	0.7882	0.8523	0.039*	
C15	1.0716 (2)	0.4885 (6)	0.82314 (17)	0.0269 (7)	
H15A	1.0466	0.4919	0.7745	0.032*	
C16	0.4073 (2)	1.3480 (6)	0.53901 (16)	0.0279 (7)	
H16C	0.3579	1.3783	0.5505	0.042*	
H16D	0.3721	1.2787	0.4904	0.042*	
H16A	0.4412	1.5211	0.5431	0.042*	
H1N2	0.852 (2)	0.242 (7)	0.7974 (17)	0.039 (10)*	
H1N1	0.651 (3)	0.424 (9)	0.775 (2)	0.078 (15)*	

### Atomic displacement parameters (Å^2^)

**Table:**

	*U*^11^	*U*^22^	*U*^33^	*U*^12^	*U*^13^	*U*^23^
S1	0.0251 (4)	0.0384 (5)	0.0310 (5)	0.0068 (3)	0.0211 (4)	0.0085 (3)
O2	0.0175 (11)	0.0343 (12)	0.0359 (12)	0.0052 (9)	0.0189 (10)	0.0070 (9)
N1	0.0166 (13)	0.0302 (14)	0.0268 (14)	0.0042 (10)	0.0157 (12)	0.0023 (10)
N2	0.0151 (12)	0.0249 (14)	0.0253 (14)	0.0030 (10)	0.0127 (11)	0.0040 (10)
N3	0.0185 (13)	0.0269 (14)	0.0266 (14)	0.0036 (10)	0.0149 (11)	0.0030 (10)
C1	0.0173 (14)	0.0322 (17)	0.0246 (16)	−0.0003 (12)	0.0140 (13)	0.0001 (12)
C2	0.0212 (15)	0.0317 (17)	0.0258 (16)	0.0013 (12)	0.0156 (13)	0.0055 (13)
C3	0.0184 (15)	0.0252 (16)	0.0234 (16)	0.0012 (12)	0.0114 (13)	−0.0001 (12)
C4	0.0180 (15)	0.0286 (16)	0.0311 (17)	0.0010 (12)	0.0163 (13)	0.0005 (13)
C5	0.0235 (16)	0.0284 (17)	0.0273 (16)	0.0017 (12)	0.0180 (14)	0.0038 (13)
C6	0.0154 (14)	0.0229 (15)	0.0214 (15)	−0.0028 (11)	0.0104 (12)	−0.0031 (11)
C7	0.0165 (14)	0.0234 (15)	0.0212 (15)	−0.0030 (11)	0.0109 (12)	−0.0029 (12)
C8	0.0173 (14)	0.0265 (16)	0.0222 (15)	0.0005 (12)	0.0116 (12)	0.0002 (12)
C9	0.0208 (15)	0.0247 (16)	0.0303 (17)	0.0046 (12)	0.0169 (14)	0.0056 (12)
C10	0.0180 (15)	0.0315 (17)	0.0238 (16)	0.0037 (12)	0.0124 (13)	0.0037 (12)
C11	0.0156 (15)	0.0248 (16)	0.0285 (16)	0.0051 (12)	0.0137 (13)	0.0030 (12)
C12	0.0216 (16)	0.0360 (18)	0.0268 (17)	−0.0014 (13)	0.0137 (14)	−0.0002 (13)
C13	0.0214 (16)	0.0293 (17)	0.044 (2)	−0.0020 (13)	0.0185 (15)	−0.0035 (14)
C14	0.0265 (17)	0.0298 (18)	0.052 (2)	0.0030 (14)	0.0292 (17)	0.0059 (15)
C15	0.0246 (16)	0.0330 (18)	0.0327 (17)	0.0063 (13)	0.0220 (14)	0.0052 (13)
C16	0.0229 (16)	0.0327 (18)	0.0286 (17)	0.0048 (13)	0.0150 (14)	0.0027 (13)

### Geometric parameters (Å, º)

**Table:**

S1—C8	1.678 (3)	C5—H5A	0.9500
O2—C7	1.228 (3)	C6—C7	1.493 (4)
N1—C7	1.376 (4)	C9—C10	1.523 (4)
N1—C8	1.397 (4)	C9—H9A	0.9900
N1—H1N1	0.80 (5)	C9—H9B	0.9900
N2—C8	1.325 (3)	C10—C11	1.519 (4)
N2—C9	1.460 (3)	C10—H10A	0.9900
N2—H1N2	0.87 (3)	C10—H10B	0.9900
N3—C11	1.335 (4)	C11—C12	1.392 (4)
N3—C15	1.351 (4)	C12—C13	1.391 (4)
C1—C2	1.376 (4)	C12—H12A	0.9500
C1—C6	1.393 (4)	C13—C14	1.374 (4)
C1—H1A	0.9500	C13—H13A	0.9500
C2—C3	1.402 (4)	C14—C15	1.386 (4)
C2—H2A	0.9500	C14—H14A	0.9500
C3—C4	1.390 (4)	C15—H15A	0.9500
C3—C16	1.502 (4)	C16—H16C	0.9800
C4—C5	1.391 (4)	C16—H16D	0.9800
C4—H4A	0.9500	C16—H16A	0.9800
C5—C6	1.399 (4)		
			
C7—N1—C8	128.8 (2)	N2—C9—H9A	109.5
C7—N1—H1N1	119 (3)	C10—C9—H9A	109.5
C8—N1—H1N1	111 (3)	N2—C9—H9B	109.5
C8—N2—C9	125.0 (2)	C10—C9—H9B	109.5
C8—N2—H1N2	113 (2)	H9A—C9—H9B	108.1
C9—N2—H1N2	121 (2)	C11—C10—C9	114.1 (2)
C11—N3—C15	117.7 (3)	C11—C10—H10A	108.7
C2—C1—C6	121.1 (3)	C9—C10—H10A	108.7
C2—C1—H1A	119.4	C11—C10—H10B	108.7
C6—C1—H1A	119.4	C9—C10—H10B	108.7
C1—C2—C3	120.9 (3)	H10A—C10—H10B	107.6
C1—C2—H2A	119.5	N3—C11—C12	122.6 (3)
C3—C2—H2A	119.5	N3—C11—C10	117.9 (3)
C4—C3—C2	117.8 (3)	C12—C11—C10	119.5 (3)
C4—C3—C16	121.4 (3)	C13—C12—C11	118.7 (3)
C2—C3—C16	120.8 (3)	C13—C12—H12A	120.6
C3—C4—C5	121.8 (3)	C11—C12—H12A	120.6
C3—C4—H4A	119.1	C14—C13—C12	119.3 (3)
C5—C4—H4A	119.1	C14—C13—H13A	120.3
C4—C5—C6	119.7 (3)	C12—C13—H13A	120.3
C4—C5—H5A	120.2	C13—C14—C15	118.4 (3)
C6—C5—H5A	120.2	C13—C14—H14A	120.8
C1—C6—C5	118.7 (3)	C15—C14—H14A	120.8
C1—C6—C7	116.4 (2)	N3—C15—C14	123.3 (3)
C5—C6—C7	124.9 (2)	N3—C15—H15A	118.4
O2—C7—N1	122.0 (3)	C14—C15—H15A	118.4
O2—C7—C6	121.0 (3)	C3—C16—H16C	109.5
N1—C7—C6	117.0 (2)	C3—C16—H16D	109.5
N2—C8—N1	115.7 (2)	H16C—C16—H16D	109.5
N2—C8—S1	126.4 (2)	C3—C16—H16A	109.5
N1—C8—S1	117.8 (2)	H16C—C16—H16A	109.5
N2—C9—C10	110.5 (2)	H16D—C16—H16A	109.5
			
C6—C1—C2—C3	−0.5 (5)	C9—N2—C8—N1	173.5 (3)
C1—C2—C3—C4	1.0 (4)	C9—N2—C8—S1	−6.9 (4)
C1—C2—C3—C16	−178.7 (3)	C7—N1—C8—N2	−4.4 (4)
C2—C3—C4—C5	−0.9 (4)	C7—N1—C8—S1	175.9 (2)
C16—C3—C4—C5	178.8 (3)	C8—N2—C9—C10	−98.0 (3)
C3—C4—C5—C6	0.4 (4)	N2—C9—C10—C11	−63.9 (3)
C2—C1—C6—C5	−0.1 (4)	C15—N3—C11—C12	−1.0 (4)
C2—C1—C6—C7	179.8 (3)	C15—N3—C11—C10	177.2 (3)
C4—C5—C6—C1	0.1 (4)	C9—C10—C11—N3	1.1 (4)
C4—C5—C6—C7	−179.7 (3)	C9—C10—C11—C12	179.4 (3)
C8—N1—C7—O2	2.1 (5)	N3—C11—C12—C13	1.5 (4)
C8—N1—C7—C6	−178.7 (3)	C10—C11—C12—C13	−176.6 (3)
C1—C6—C7—O2	0.8 (4)	C11—C12—C13—C14	−0.5 (4)
C5—C6—C7—O2	−179.3 (3)	C12—C13—C14—C15	−0.9 (4)
C1—C6—C7—N1	−178.3 (3)	C11—N3—C15—C14	−0.5 (4)
C5—C6—C7—N1	1.5 (4)	C13—C14—C15—N3	1.5 (4)

### Hydrogen-bond geometry (Å, º)

**Table:**

*D*—H···*A*	*D*—H	H···*A*	*D*···*A*	*D*—H···*A*
N2—H1*N*2···O2	0.87 (4)	1.90 (3)	2.645 (3)	143 (3)
N2—H1*N*2···N3	0.87 (4)	2.41 (4)	2.860 (4)	113 (3)
C1—H1*A*···O2	0.95	2.42	2.751 (4)	100
C9—H9*B*···S1	0.99	2.72	3.166 (4)	108
C14—H14*A*···O2^i^	0.95	2.51	3.421 (4)	161

Symmetry code: (i) −*x*+2, *y*+1/2, −*z*+3/2.
